# Editorial to “investigating the role of electroanatomical mapping in single‐shot pulsed field catheter ablation”

**DOI:** 10.1002/joa3.13184

**Published:** 2024-11-08

**Authors:** Yoshiaki Mizutani, Satoshi Yanagisawa, Yasuya Inden

**Affiliations:** ^1^ Department of Cardiology Yokkaichi Municipal Hospital Yokkaichi Mie Japan; ^2^ Department of Cardiology Nagoya University Graduate School of Medicine Nagoya Aichi Japan

Atrial fibrillation (AF) is a common cardiac arrhythmia that is associated with an increased risk of stroke, heart failure, dementia, and mortality. Pulmonary vein isolation (PVI) is an effective rhythm control strategy for treating AF.^1^ Safe and effective treatments for PVI have been established with cryoballoon and radiofrequency ablation, both of which use thermal energy in the myocardium. Meanwhile, pulsed field ablation (PFA), a novel ablation technology that uses non‐thermal energy, provides electrical pulses to cause non‐thermal irreversible electroporation and induce cardiac cell death.

In this issue of the *Journal of arrhythmia*, Kariki et al.^1^ compared the arrhythmia recurrence and procedural characteristics of PFA for AF between the electroanatomical mapping (EAM) and fluoroscopy groups. Fifty‐one patients with AF who underwent PVI for the first time were included in their study (fluoro‐only group, 31 patients; EAM group, 20 patients). PVI was performed using the FARAPULSE™ PFA system (Boston Scientific, Natick, MA, USA). In the EAM group, the ablation catheter was exchanged with a multipolar mapping catheter (Advisor™ HD Grid catheter [Abbott Laboratories, Abbott Park, Ill, USA]) after PVI, and the EAM of the left atrium was subsequently generated. As a result of the acute procedures, the procedure time was significantly longer in the EAM group than in the fluoroscopy‐only group, whereas there was no significant difference in the fluoroscopy time between the two groups. During a mean follow‐up period of 11.2 months, PVI with EAM did not lead to significantly different arrhythmia recurrence rates compared with PVI without EAM. No complications were observed in either of the groups.

In their study, EAM was performed only after fluoroscopy‐guided PFA and not before ablation in the EAM group. Obtaining an EAM with a mapping catheter preoperatively is useful for understanding the pulmonary vein (PV) and left atrial anatomy, which may support the learning curve for PV identification and increase the certainty of catheter manipulation, especially for young fellows and residents. Perhaps the acquisition of EAM prior to ablation, in addition to post‐mapping, may have influenced the procedure outcomes differently, despite the small number of samples in the current study. A recent non‐randomized study with 197 patients undergoing first PVI using the same PFA catheter at a tertiary referral center reported the same efficacy of recurrence‐free rate after a 12‐month follow‐up between the pre‐ and post‐mapping group (*n* = 127) and non‐mapping group (*n* = 70), and the median procedure duration, left atrial dwell time, and fluoroscopic time were significantly shorter in the non‐mapping group than in the mapping group.^2^ These findings suggest that the creation of a preoperative EAM is unnecessary. Similarly, the current study demonstrated several possible advantages of skipping the creation of perioperative EAM during PFA. First, the procedural time can be reduced substantially. Second, there is concern that changing catheters from a relatively thick guiding sheath might cause air emboli, and not switching to another mapping catheter can reduce the risk of microair‐emboli.^3^ Furthermore, the FARAWAVE PFA catheter has an electrode in each spline that can generate an EAM on a 3‐dimensional mapping system, and the presence/absence of PV potentials and conduction velocity can be identified despite the small number of acquiring points and relatively low resolution of the mapping image. In addition, the absence of PV potentials after PFA in the acute phase may not always be associated with durable lesion formation, because insufficient electrode contact of the PFA catheter could create a relatively large reversible zone surrounding the irreversible lesion and transient conduction block, which cannot be classified based on post‐mapping immediately after ablation.^4^ Finally, ablation procedures without using mapping catheters can reduce medical costs.

PVI is the cornerstone of AF ablation strategies. However, it is known that paroxysmal AF and persistent/long‐standing persistent AF have different recurrence rates after PVI only.^5^ In the current study, both groups included a mixture of patients with PAF and non‐PAF. It is not uncommon for the anatomy of the left atrium and PVs to be deformed in patients with persistent AF, and a larger isolation area covering the PVs, left antrum, and damaged substrate is expected to suppress the incidence of repeated AF. However, differences in the patient sample AF types were not statistically significant between the groups in this study. It would be interesting to examine the usefulness of EAM evaluation, focusing on patients with persistent AF. Moreover, the small samples of only five residual PV potentials (6.3%) in the EAM group might underpower the discrimination of the difference in prognosis, given that other factors of non‐PV foci and emerging atrial tachycardia/flutter may affect the recurrence rate in addition to PV reconnection, and the authors did not report the prognosis of recurrence in patients receiving additional PFA for the five residual PV potentials. Further systematic evaluations in randomized controlled studies with large‐scale samples are required.

## FUNDING INFORMATION

This research did not receive any specific grants from funding agencies in the public, commercial, or not‐for‐profit sectors.

## CONFLICT OF INTEREST STATEMENT

Authors declare no conflict of interests for this article.
